# Strategies for Bone Regeneration: From Graft to Tissue Engineering

**DOI:** 10.3390/ijms22031128

**Published:** 2021-01-23

**Authors:** Giulia Battafarano, Michela Rossi, Viviana De Martino, Francesco Marampon, Luca Borro, Aurelio Secinaro, Andrea Del Fattore

**Affiliations:** 1 Bone Physiopathology Research Unit, Genetics and Rare Diseases Research Division, Bambino Gesù Children’s Hospital, IRCCS, 00146 Rome, Italy; giulia.battafarano@opbg.net (G.B.); michela1.rossi@opbg.net (M.R.); 2 Department of Clinical, Internal, Anesthesiology and Cardiovascular Sciences, “Sapienza” University of Rome, 00161 Rome, Italy; viviana.demartino@uniroma1.it; 3 Department of Radiotherapy, “Sapienza” University of Rome, 00161 Rome, Italy; francesco.marampon@uniroma1.it; 4 Advanced Cardiovascular Imaging Unit, Department of Imaging, Bambino Gesù Children’s Hospital, IRCCS, 00165 Rome, Italy; luca.borro@opbg.net (L.B.); aurelio.secinaro@opbg.net (A.S.)

**Keywords:** bone regeneration, tissue engineering, scaffolds, mesenchymal stem cell, bio-materials

## Abstract

Bone is a regenerative organ characterized by self-renewal ability. Indeed, it is a very dynamic tissue subjected to continuous remodeling in order to preserve its structure and function. However, in clinical practice, impaired bone healing can be observed in patients and medical intervention is needed to regenerate the tissue via the use of natural bone grafts or synthetic bone grafts. The main elements required for tissue engineering include cells, growth factors and a scaffold material to support them. Three different materials (metals, ceramics, and polymers) can be used to create a scaffold suitable for bone regeneration. Several cell types have been investigated in combination with biomaterials. In this review, we describe the options available for bone regeneration, focusing on tissue engineering strategies based on the use of different biomaterials combined with cells and growth factors.

## 1. Bone Biology: Structure and Composition

Bone tissue has been assigned to give support to muscle, allow movement and locomotion, to provide organs with protection, to host bone marrow, and to regulate mineral homeostasis and the endocrine functions of the body, such as glucose tolerance, insulin sensitivity and cognitive behaviors [1].

According to their shape, four types of bones can be anatomically classified as long, short, flat, and irregular bones. Bone tissue is organized in a hierarchical structure. At the macroscopic level, we can identify two kinds of structures: cancellous (also called trabecular or spongy bone) and cortical (or compact) bone. While cortical bone corresponds to around 80% of the total amount of bone of the skeleton, the trabecular bone constitutes the remaining 20%. All bones are composed of both cancellous and cortical bone, present in different and specific percentages for each type of bone. For example, at the diaphysis of long bones, a thick cortical bone is observed that moves longitudinally to the epiphysis and becomes a thin shell covering the cancellous bone; flat bones like the calvaria have a sandwich structure with a dense cortical bone on the outside and interior surfaces of cancellous bone [2].

The cortical bone appears as a dense outer bone surrounding the marrow space, whereas the trabecular bone consists of inner trabecular plates and rods, building a honeycomb-like network in the marrow compartment [3]. These two kinds of bone also differ in their levels of tissue porosity and metabolism. Trabecular bone is more porous (with a porosity ranging from 40% to 95% [4]) and less dense than cortical bone, giving this spongy bone a more flexible and weaker structure. Moreover, cancellous bone is more metabolically active than compact bone. Thus, the structure reflects the function.

At the microscopic level, in the cortical bone, the osteon represents a cylinder running parallel to the longitudinal axis of the bone. This structure of about 200 μm of diameter, contains concentric layers of lamellae wrapped around a central canal [2]. In cancellous bone, the lamellae organize to build a framework consisting of rods and plates of trabeculae. Each rod is about 50–300 μm in diameter [2]. The trabeculae arrange themselves in the direction of mechanical loads, conferring resistance in a different way compared to compact bone. The sub-microstructure is composed of lamellae consisting of sheets of fibers of mineralized collagen arranged in a planar structure. Within collagen fibers, collagen fibrils are made of hydroxyapatite (HA) mineral crystals and collagen molecules [2]. The crystals are approximately 3 × 25 × 50 nm in size, and are either intra- or extra-fibrillar [5]; intra-fibrillar crystals are associated with the gap regions of the collagen fibril [6], while extra-fibrillar crystals are found in the space surrounding the fibrils [7].

The mineralized extracellular matrix identifies bone tissue as specialized connective tissue. Indeed, at the molecular level, the bone extracellular matrix consists of an organic phase, made of about 90% type I collagen and of approximately 5% non-collagenous proteins, an inorganic phase constituted by hydroxyapatite, and water (10–20%) [8].

Several types of cells are hosted in the tissue: mesenchymal stem cells (MSC), bone-forming osteoblasts and osteocytes, derived from mesenchymal lineage, bone-resorbing osteoclasts originating from hematopoietic lineage and immune cells [1]. Indeed, bone and immune cells share the same microenvironment and they regulate each other [9]. T cells regulate osteoclasts and, in particular, the Th17 population represents the most osteoclastogenesis-inducing variety of T cell, while T regulatory cells exert an inhibitory effect on osteoclast differentiation and activity [9]. Moreover, B cells produce osteoclastogenic cytokines [10] and neutrophils and natural killer cells have been also involved in bone homeostasis regulation particularly in inflammation mediated bone loss and autoimmune diseases [9].

MSC represent the adult osteo-progenitor stem cell population located in bone. As stem cells, MSC are defined by two properties: clonogenic self-renewal and multi-potential lineage differentiation. Indeed, stem cells can undergo asymmetric division to produce one cell identical to the parent which continues to contribute to the original stem cell line, and one with a reduced proliferative capacity and more restricted developmental potential in comparison to its parent [11]. The current concept of mesenchymal stem cells can be traced back to their first observation, dating back to 1869. This involved the realization that the transplantation of bone marrow into heterotopic anatomical sites resulted in the de novo generation of ectopic bone [12]. After proof of the osteogenic potential residing in bone marrow was demonstrated by Tavassoli and Crosby [13], MSC were first isolated as a subpopulation of marrow cells by Friedenstein and collaborators between 1960–1990 [14,15,16]. MSC have been defined by the consensus position statement of The International Society for Cellular Therapy, as a population of multipotent non-hematopoietic stromal cells derived from bone marrow and other mesenchymal tissues that can be isolated by their ability to adhere and grow on a plastic surface in vitro. These cells exhibit a specific pattern of positive and negative surface markers and have the ability to differentiate into osteogenic, chondrogenic and adipogenic lineage [17]. Indeed, MSC are characterized by high levels of expression of CD73, CD90 and CD105 on their membrane surface and yield negative results for CD45, CD34, CD14, CD19, CD11b and HLA (Human Leukocyte Antigen)-DR [17]. So far, MSC have been identified in several fetal and adult tissues beyond the bone marrow, such as adipose, muscle, blood, placenta, dental pulp and umbilical cord tissues [18].

Osteoblasts represent 4–6% of the total resident cell population of bone cells and are largely known for their bone-forming function. They derive from MSC that, after committing towards osteogenic lineage, differentiate into osteoblast progenitors expressing *Runx2* (Runt-related transcription factor 2) and *Col1a1* (Collagen 1a1) genes. Then, the pre-osteoblasts evolve into mature osteoblasts that undergo morphological changes becoming large and cuboidal cells. This transition is related to bone matrix synthesis that occurs in two steps: the deposition of an organic matrix made by collagen and non-collagenic proteins, followed by its subsequent mineralization with the vesicular and fibrillar phases [1]. At this stage, the mature osteoblasts can undergo apoptosis or become osteocytes or bone lining cells [1].

Osteocytes represent the last differentiation state of osteoblasts which remain embedded in the bone matrix, residing in lacunae. They were historically described as quiescent cells entrapped in the bone matrix; actually, osteocytes are recognized as key cells for normal skeletal functions, playing a critical role in bone homeostasis maintenance, acting as mechanosensors, regulating phosphate homeostasis through secretion into the circulation of fibroblast growth factor 23 (FGF23) [19] and orchestrating the bone remodeling process via the direct regulation of both osteoblast and osteoclast activities [19].

Osteoclasts are polarized giant cells responsible for bone resorption which occurs through the dissolution of the inorganic phase of the bone matrix and the degradation of collagen proteins [1]. They are defined as multinucleated tartrate resistant acid phosphatase (TRAP) positive cells originating from monocyte-macrophage lineage. Indeed, they differentiate from the fusion of mononuclear cells of hematopoietic lineage origin, under the influence of several factors, such as macrophage colony-stimulating factor (M-CSF) and RANKL (receptor activator of nuclear factor κ B ligand). Particularly, RANKL is expressed by osteoblasts, stromal cells and immune cells as a membrane-bound form or released as a cytokine; it binds to its receptor RANK on osteoclast precursors and osteoclasts, regulating their differentiation, survival and activity [20]. In the RANKL/RANK interaction, the cytokine osteoprotegerin (OPG) exerts a pivotal function since it is a soluble decoy receptor for RANKL, preventing its binding to RANK, inhibiting osteoclastogenesis and thus protecting from excessive bone resorption [21]. Consequently, the RANK/RANKL/OPG triad is very important for the regulation of bone remodeling. Indeed, despite the inert appearance, bone is a very dynamic tissue subjected to a continuous “building–destroying” cycle called remodeling. The remodeling is a complex process by which old micro-damaged bone is replaced with new formed bone. The remodeling cycle is made up of three principal phases: the osteoclast-mediated bone resorption phase, during which both mineral and collagenous matrixes are dissolved; the reversal period, that matches the time and space of the resorption process to the formation of new tissue; and finally, the new deposition of the bone matrix made by the osteoblasts [22,23,24]. The tight coupling of osteoblast and osteoclast activities guarantees the correct spatial and temporal recruitment of cells only in the site to be replaced, in order to maintain bone structure [25]. When the process is un-coupled and the phases succeed without the correct combination of time and site, pathologic conditions occur. The bone remodeling process clearly demonstrates that bone is a regenerating organ.

## 2. Bone Regeneration: Replacement and Tissue Engineering

### 2.1. Natural Grafts Versus Synthetic Grafts

Bone is a unique tissue that continuously and completely regenerates. In the clinical setting, the most common evidence of bone regeneration is fracture healing [26].

There are two types of fracture healing: primary (or direct) fracture healing and secondary (or indirect) healing. The first one requires a stable fixation and a correct anatomical reduction in fracture ends; it can occur by the direct remodeling of the lamellar bone [27].

**Figure 1:**
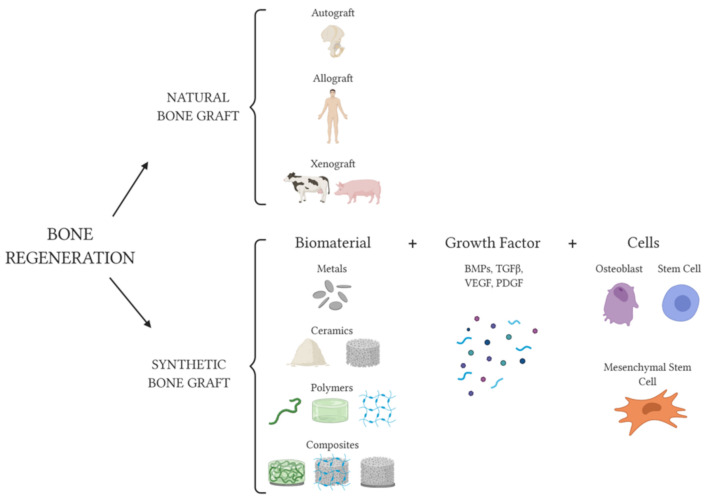
Representative scheme of available options for bone tissue regeneration. Natural bone grafts include autograft, allograft from human donors and xenograft from other species. Synthetic bone grafts can be obtained using metals (gray discs), ceramics (beige powder and gray microporous cylinder-shaped object) and polymers (green ribbon, green stiff cylinder-shaped hydrogel, light blue hydrogel mesh). Biomaterials can be functionalized with growth factors such as BMPs (bone morphogenetic proteins; BMP-2, BMP-7, BMP-9 here represented as blue and green circles and violet hexagon), TGFβ (transforming growth factor beta; pink hexagon), VEGF (vascular endothelial growth factor; blue ribbon) and PDGF (platelet-derived growth factor; light blue ribbon). Moreover, synthetic bone graft can be colonized by cells. The figure was created using BioRender (https://biorender.com/).

Indirect fracture healing consists of both endochondral and intramembranous bone healing; it does not require anatomical reduction or rigidly stable conditions. Indirect bone healing occurs in non-operative fracture treatment and in the external/internal fixation of complicated comminuted fractures. After trauma, an inflammatory phase proceeds. Indeed, acute inflammation that peaks after 24 h, recruits cells from both the peripheral and intramedullary blood. After the hematoma formation, the MSC recruitment occurs leading to the generation of a soft callus made of cartilaginous tissue. Subsequently, neo-angiogenesis and revascularization allow for the transformation of a cartilaginous matrix into an osseous tissue and the primary soft callus can be replaced by a hard bony callus. Finally, since the hard callus is made of woven bone, its remodeling process, carried out by osteoclasts and osteoblasts, allows for a substitution with the lamellar bone. Thus, the newly formed tissue is eventually indistinguishable from the adjacent uninjured bone [26,27,28]. However, bone renewal is sometimes impaired, for example, in delayed union and non-union fractures that can occur when there is an unstable fixation. In addition, there are cases in which the regenerative demand is beyond the normal potential for self-healing, such as in critical-size bone defects derived from orthopedic or oral-maxillofacial surgeries following traumas, infections and tumor resections [26]. In all of these cases, clinical intervention is needed to exogenously regenerate the bone tissue. There are three main elements required for bone tissue regeneration: cells, growth factors/morphogenic signals, and a scaffold material which supports them (Figure 1). Actually, two options of scaffolds are available for treating bone defects: natural or synthetic bone-grafts. Natural grafts can be classified by their origin in autografts, allografts or xenografts (Figure 1). The former is a graft which is provided in an autologous manner and is the preferred method for clinical practices, particularly to treat large bone defects [29,30,31]. Even though autologous grafts may be harvested from several skeletal sites, the iliac crest has so far been the “gold standard” source for surgical procedures due to the relatively high amount of available bone [29,30,32]. Autografts are free from risks of disease transmission and autoimmune rejection [32]. Moreover, they retain the same regeneration and remodeling properties of the living bone of the host [32]; however, their use is limited due to morbidity at the harvesting site leading to various complications, including infection, haematoma/seroma, fracture, nerve and vascular injuries, chronic donor site pain, hernias, unsightly scars and to restricted achievable quantities [33]. For allografts, the bone graft is taken from a donor and can be obtained in greater quantities compared to the autograft. Due to the standard protocols of harvesting, collection and storage, the risk of disease transmission is negligible; however, these grafts integrate more slowly and to a lower degree than the autografts [34]. Xenografts, or heterologous grafts, derive from other species, commonly porcine and bovine sources [35]. The availability of xenografts is theoretically unlimited when correctly processed to be safe for the host, despite the potential transmission of zoonosis [35]. Both allografts and xenografts undergo acellularization to decrease antigenicity, thus resulting in them belonging to the tissue engineering category [35]. Synthetic bone-grafts are classed as biomaterials, substances engineered to interact with living systems and intended for tissue replacement, that can be classified depending on their composition in metals, ceramics, polymers and composites (Figure 1) [36].

### 2.2. Features of a Biomaterial for Bone Tissue Engineering

For application into a living organism, such as humans, all materials must have specific features. First of all, to be defined as a biomaterial, the material needs to be biocompatible and therefore to exist in harmony with the host’s biological fluids, tissues and cells, without causing harmful effects locally or systemically. Moreover, the chemical features influence the host cell’s behavior as well as the surface topography and wettability, necessary for allowing cell adhesion, proliferation and differentiation. The three-dimensional configuration is also an important parameter. Indeed, it must provide a structure for the new formation of tissue in a 3D manner. In addition, porosity, pore size and shape are critical for tissue engineering. Pores must allow cell colonization and vascularization. The pore size should be maintained within the range of 200–350 μm to guarantee cell ingrowth [37]. In order to develop a scaffold to substitute a tissue, specific mechanical properties of the biomaterial must mimic, as much as possible, those of the tissue that it is attempting to resemble. In the case of bone, viscoelasticity and shear stress are essential parameters to consider [31]. Moreover, when the aim is also to achieve the regeneration of the tissue and the complete substitution of exogenous material with new healthy physiological tissue, another important criterion is resorbability [35]. Specifically referring to the regeneration of bone, three features need to be mentioned: osteoinduction, osteoconduction and osteointegration. Indeed, the aim of the exogenous material used to replace bone is hidden in these words. Osteoinduction means the ability to induce osteogenic differentiation of a cell that is not yet committed [38]. Thus, an osteoinductive material is something that can directly induce osteogenesis through the recruitment, proliferation, and differentiation of mesenchymal stem cells [39]. Osteoconduction is the capacity to provide the micro-environment to allow the occurrence of orthotopic osteogenesis. So, an osteoconductive material allows bone growth on it. Osteointegration was firstly described by Brånemark and co-workers [40], who observed via light microscopy, a direct contact between titanium implants and bone. Then, osteointegration was histologically defined by Dorland’s Illustrated Medical Dictionary as the direct anchorage of an implant to the living bone by bony tissue and without the growth of fibrous tissue at the bone–implant interface [41].

### 2.3. Metals

The first materials used in bone grafts were metals such as Fe, Mg, Zn and their alloys [31]. The use of an iron-made dental implant in a man living during the end of the first century AD has even been reported [42]. Magnesium-based implants have also been explored as biocompatible and degradable implants for load-bearing applications [43]. Zhao and co-workers demonstrated the usefulness of biodegradable Mg screws for the stabilization of the bone flap in patients with osteonecrosis of the femoral head [44]. Non-degradable metallic biomaterials have been developed for the fabrication of implants, such as artificial hip joints and bone plates beyond dental permanent applications. Examples of these metals are Ni-free stainless steel, titanium, and its alloys composed of non-toxic and allergy-free elements and Co-Cr alloys [45].

### 2.4. Ceramics

Ceramics are inorganic non-metallic materials [46]. Since 70% of bone tissue is made up of hydroxyapatite, biomaterials such as calcium phosphate ceramics (CaPs) and bioactive glasses (BG) were introduced more than 30 years ago and are still used as bone substitutes [47]. Their bioactivity derives from their mimicry of the mineral phase of bone, thus providing a suitable surface for new tissue formation [47,48]. Mechanical properties of ceramics have been reported; even though ceramics have a higher mechanical strength than human cortical bone, they show lower toughness and a higher Young’s modulus than those of cortical bone, making them undesirable for the repair of load bearing bones [48].

The most common CaPs used in bone tissue engineering are hydroxyapaptite, tricalcium phosphate (TCP) and a combination of the two, known as biphasic calcium phosphate (BCP). They have a composition similar to that of natural bone, good biocompatibility, osteoconductivity and can osteointegrate [49]. TCP exists in two major distinct phases of crystals (alpha and beta) similar in their chemical composition but differing for their crystallographic features that confer them different resorption features [50]. While HA has a relatively high crystallinity and it is difficult to degrade in vivo, TCP is more degradable than HA and becomes soluble more rapidly [51].

Calcium phosphate opened the way for cement development being the first injectable biologic cement approved by the FDA in 1998 [52]. To date, many injectable types are used in clinical practice to treat bone defects [31] and are commercially available in several forms [53,54,55,56,57]. Bioactive glasses, silicate glass-based materials, are osteoconductive and osteoinductive in certain formulations. Moreover, by varying the proportion of sodium oxide, calcium oxide and silicon dioxide, all types of soluble and non-resorbable forms can potentially be produced [31]. The interest in bioactive glasses is due to their ability to bond with both bone and soft tissues [58]. Following the implantation of the scaffold, their interfacial bond induces the formation of a dense layer of hydroxyapatite carbonate very similar to the bone mineral phase, allowing cell adhesion [48]. Zhang and colleagues demonstrated that bioactive borate glass shows better performance than TCP [59]. In the elegant review by El-Rashidy and co-workers, it was reported that the regenerative capacity of the BG scaffolds depends on various factors such as composition, microstructure, and fabrication methods. Indeed, based on differences in composition and proportion, bioactive glasses can be classified in 45S5, 58S and 1393 categories [49].

### 2.5. Polymers

Polymers can be natural or synthetic materials. Among the natural polymers there are collagen, chitosan, alginate, elastin and cellulose; all of them have been applied for regeneration and tissue engineering purposes. Natural polymers are considered as the first biodegradable materials that offer the possibility of being completely substituted by new bone [35]. Their resorbability is based on their enzymatic or hydrolytic degradation [60].

Collagen is the most widely used biomaterial not only for medical application but also for biomedical research purposes [61]. Reasons for this include the fact that it is a major component of the extracellular matrix, it is a non-toxic material that is easy to isolate from various tissue sources, and it has high a level of biocompatibility and very low immunogenicity [61]. In contrast, pure type I collagen has more variability in terms of cross-link, fiber size, density and impurities compared to isolated collagen. Moreover, its hydrophilicity can lead to swelling [62,63].

Chitosan is a biodegradable and biocompatible natural polysaccharide adopted in a wide range of fields such as pharmaceutics, biomedical, cosmetics, textile and the food industry [64]. It is used to allow blood clotting and wound healing because of its bonding nature, antifungal, and bactericidal properties and oxygen permeability [65,66]. It has also been reported that, depending on its formulation and structure, chitosan can support osteoblast survival, proliferation and maturation in vitro [67]. Moreover, chitosan enhances mineralization observed during the osteoblast differentiation of human bone marrow-derived mesenchymal stem cells (BM-MSC) [68]. However, it has been associated with allergic reactions and is characterized by low solubility [35]. For these reasons, chitosan has been used in combination with other biopolymers and ceramics for the engineering of tissues including cartilage, bone, skin and blood vessels, and for corneal regeneration, as accurately described by Islam and collaborators [69].

Alginate is an anionic polysaccharide derived from algae, known for its great capacity to absorb water, with the ability to absorb 300 times its own weight [35]. Beyond its biodegradable nature, it is easy to mix, manipulate and use. In particular, it has been investigated for its gel forming properties leading to the development of combined gels with other types of polymer, collagen, ceramics and bioglasses [70]. Ghosh and colleagues created a composite hydrogel with a nanofibrous structure mimicking the natural bone extracellular matrix, showing excellent mechanical properties and allowing the in vitro adhesion and viability of pre-osteoblasts [71]. Although alginate is not expensive, it shows poor dimensional and mechanical stability [35].

Synthetic polymers represent an attractive solution because of their physicochemical and mechanical properties. Non-toxic and FDA approved synthetic polymers include: PLA (polylactic acid) and PGA (polyglycolic acid), showing the best mechanical properties, being non-inflammatory, biocompatible and biodegradable and supporting cell adhesion [72,73]; biodegradable PLGA (polylactic co-glycolic acid), providing support for cell adhesion; and PCL (poly e-caprolactone), which shows a relatively slow degradation rate and a great compatibility with human MSC [74]. PEG (polyethylene glycol) is another synthetic polymer that is very widely used, not only in tissue engineering but also in pharmacy, industrial chemistry, medicine and biology.

Among the polymers that show high mechanical strength there are PBT (polybutylene terephthalate) and PET (polyethylene terephthalate), both of them are highly biocompatible, biodegradable and impact resistant [75,76]. However, numerous studies have highlighted the troublesome effects of PET on the endocrine system and, thus, its use in biomedical applications needs further investigation [77,78,79]. Since PPF (polypropylene fumarate) is biocompatible and has good mechanical properties with a suitable decomposition rate, it has been used for biomedical engineering and orthopaedic applications [80]. Finally, PAA (polyacrylic acid) warrants mentioning in terms of its application in permanent implants, since it is not biodegradable [81].

### 2.6. Advantages and Disadvantages of Materials for Bone Tissue Engineering

Each biomaterial has its advantages and disadvantages. First of all, the best osteoinductive materials are the grafts and in some cases ceramic materials, such as hydroxyapatite [82]. Beyond the osteoinductive and osteoconductive properties of grafts, the other advantages of using them include their mechanical properties, such as a Young’s modulus similar to native bone, biocompatibility and perfect osteointegration. However, some disadvantages underlie the requirement of other solutions in order to treat bone lesions, for example, the limited availability of grafts, the costs and the impossibility to create a personalized product [31]. In the case of allo- and xenografts, the eventual rejection, the risk of disease transmission, the possibility to lose parts of their osteoinductive properties due to the processing required to counteract their antigenic features, and the associated ethical concerns need to be considered [31,35].

The use of synthetic bone graft allows for the personalization of the product in the form of adapting the scaffold to the patient’s anatomy. Regarding metals, they show excellent mechanical properties, are biocompatible and allow for osteointegration. On the contrary, the higher stiffness of the implant can lead to stress shielding resulting in bone loss and increasing the risk of implant loosening [83]. Moreover, there is a risk of corrosion and the release of toxic metal ions related to their use in biological systems [31]. Ceramics also show good mechanical properties and are not related to risk of toxicity since they have excellent resistance to corrosion. However, they are brittle with low plasticity [31] and often show insufficient degradability [35]. Polymers are the best choice for patient-customized scaffold development and are biocompatible, but their mechanical properties depend on their formulation. Indeed, they have a lower Young’s modulus than ceramics and metals [31]. However, since this class encompasses several different biomaterials, various specific mechanical properties can be associated with each polymer. Since their resorbability can be controlled during production, polymers offer the best grade of biodegradability both in the cases of natural polymers and synthetic compounds [35].

Polymers, as personalizing materials, also offer the possibility of modifying their features, such as shape and porosity, by using different fabrication methods. For example, using a melt extrusion technique Evans and colleagues obtained a surface-porous polyetheretherketone (PEEK-SP) that—compared to the non-porous controls—showed improved osteointegration in a rat model; moreover, the structural integrity necessary for load-bearing was maintained [84].

Although synthetic bone grafts overcome the problem of personalization that is missing with natural grafts, they show the important bias that occurs regarding the inadequate vascularization and, consequently, poor nutrient transport into the inner space of the implant. Further investigation is needed to solve this issue. In conclusion, to date, no material alone has the desired properties to efficiently resemble the mechanical competent bone tissue. For this reason, most research is focusing on the development of combined materials, functionalized with molecules and cells in order to generate new scaffolds implemented in their features.

### 2.7. Composite Materials

Composites are defined as scaffolds made of two or more substrates belonging to the same or different class of materials. Composites of metals and ceramics have been developed to counteract the corrosion of metals, without renouncing their optimal mechanical properties [55,85,86]. Kihlström Burenstam Linder et al. demonstrated how titanium-reinforced tailor-made CaP-based cranial implants can promote in situ bone regeneration and osseointegration [87], overcoming the limits of higher infection rates that are observed in cranioplastic approaches by using only cement [88].

Hydroxyapatite and alumina allow for the successful formation of new bone in vivo [89]. When fabricated by chemical crosslinking with glutaraldehyde, chitosan and gelatin, they form a gel-like structure with interconnected pores, supporting the adhesion, infiltration, proliferation and function of both pre-osteoblastic cells and human BM-MSC [90].

Other composites have also been developed using different materials belonging to the same family. BCP, a combination of HA and TCP, has been developed to increase the degradability of the scaffold [51] and to control the rate of solubility that is influenced by the calcium phosphate ratio [91].

Composites of metals and polymers have also been investigated, revealing that chitosan-TiO_2_ nanotube scaffolds with Ca^2+^ ions promote adhesion, proliferation and early differentiation of the osteosarcoma cell line [92].

Since type I collagen and biological apatite are the main constituents of physiological bone tissue, the best choice to mimic the natural functions of bone is represented by composites of polymers and ceramics such as nano-hydroxyapatite/poly-ε-caprolactone [93,94], chitosan/calcium phosphate [95] and chitosan, polyphosphate and pigeonite [96]. HA has been used to improve the mechanical properties of polymers such as PLA [97], as well as bone cell attachment and maturation on these surfaces [98,99]. Several authors have developed combined scaffolds of calcium phosphate and hydroxyapatite with collagen, alginate and chitosan [100,101,102,103,104,105]. In this context, collagen has been extensively used with TCP and HA for both in vitro and in vivo studies, supporting both woven and lamellar bone formation [106]. The addition of collagen to porcine graft and hydroxyapatite/tricalcium phosphate increases bone formation in critical size defects in rabbit calvarias [107]. Injectable collagen/α-tricalcium phosphate cement has been demonstrated to be able to give rise to a composite supporting in vitro cell adhesion and proliferation [108]. Composites of bovine type I collagen and hydroxyapatite supported the attachment and proliferation of mouse MSC and human periodontal ligament stem cells [109]. Marine spongin demonstrated the ability to support bone regeneration when used in vivo as an additive to hydroxyapatite, accelerating material degradation and enhancing the formation of new bone [110]. However, in a preclinical model of a critical size defect, a bioceramic composed of dicalcium phosphate and hydroxyapaptite was shown to be more effective than an implant made of biphasic calcium phosphate with collagen, in terms of implant stability and the percentage of marginal bone covered. This type of implant also showed qualities including optimal compression strength, early resorption of material and osteoblastic bone formation [111].

In addition to combining materials to form composites, their fabrication methods can also influence the outcomes. Indeed, El-Fiqi et al. created a novel bone-mimetic nanohydroxyapatite/collagen porous scaffold from a surface silanized mesoporous nanobioglass hybrid scaffold. The presence of nanobioglass in the fibrillar network of collagen improves the growth of HA crystals, which maintains the porosity of the collagen scaffold. Through this approach, the authors demonstrated that the mineralized scaffold possesses excellent osteogenic potential in vivo for the healing of a critical-sized calvaria bone defect [112].

All of these studies—and many others—conducted within the research field of bone regeneration, have led to several composite material products having been released onto the market and to the development of innovative design methods (e.g., TPMS). However, no such scaffold has yet been unequivocally demonstrated as being capable of resembling the structural features of bone in terms of vasculature formation, resorbability, complete substitution with new bone tissue, and organ regeneration. Thus, most research has focused on the addition of active biomolecules and/or cells to the scaffolds to recapitulate the signaling cascades that occur in physiological tissue regeneration.

### 2.8. Scaffold Functionalization

Scaffolds can be functionalized to improve their properties, for instance to reduce corrosion and increase bioactivity. When implants of magnesium are covered by bioactive ceramics, the amount of magnesium ions released into the blood plasma decreases [55]. The coating allows for reductions in the corrosion rate, improving new bone formation and reducing the inflammatory response at the implant–host tissue interface in vivo [52]. A titanium alloy, modified in its inner surfaces with a polydopamine-assisted biomimetic hydroxyapatite coating, improves the osteointegration of the scaffold and enhances attachment and proliferation of the osteoblast cell line MC3T3-E1, while also promoting bone regeneration in a condylar defect in vivo [85]. Additionally, a porous Ti-6Al-4V scaffold was fabricated with the addition of a bioactive coating in order to overcome the bio-inertness of the Ti-6Al-4V and reach the desired surface, while maintaining osteogenic ability. The scaffold obtained allowed for the attachment, proliferation, and differentiation of BM-MSC to a greater degree than was achieved by the conventional bioactive glass (BG)-coated Ti-6Al-4V scaffolds and bare-metal Ti-6Al-4V scaffolds [113]. Furthermore, Fe/Mn incorporation amplified the osteogenic promotion induced by intrafibrillar mineralized collagen-HA-based scaffolds [114]. To promote cell attachment to the biopolymers, the incorporation of a tri-amino acid sequence, arginine-glycine-aspartate, or “RGD”, onto polymeric materials has been tested [115]. Indeed, it was demonstrated that enrichment of PCL with RGD residues promotes osteoblast attachment and enhances BM-MSC proliferation and differentiation [116,117,118]. Since PEG is biologically inert, it can be functionalized with arginine-glycine-aspartate peptides in order to increase its bioactivity and allow cell adhesion [80,119,120]. Moreover, PPF immobilized arginine-glycine-aspartate residues regulate osteoblast migration [121].

Due to the relevance of vascularization in tissue regeneration, many approaches have focused on scaffold functionalization in order to promote angiogenesis. Beyond the control of scaffold microstructure and properties such as porosity to support neo-vascularization, the delivery of angiogenic molecules through biomaterials represents a strategy to promote angiogenesis. Representative angiogenic molecules include metallic ions and growth factors [122].

Metallic ions include Cu, Co, Silicate, Zn and Mg. The addition of copper to a mesoporous bioactive glass scaffold is able to improve the angiogenesis and osteogenesis of MSC [123]. Cobalt is also able to induce angiogenesis and promote osteogenesis, as demonstrated by Quinlan et al. [124]. Since silicon is an essential element for the mineralization of osteoblasts [125], it has been tested in various silicate-based biomaterials for bone regeneration purposes [126]. The addition of zinc silicate to composites of collagen and hydroxyapatite enhanced in vivo bone angiogenesis, modulating monocytes and creating a favorable osteogenic microenvironment [127]. Promising results have been also reported with nanocomposites of zeolite and collagen [128]. A scaffold can be also functionalized with growth factors that, when combined with the biomaterial, can directly reach the target site. Thus, local delivery can permit the avoidance of problems linked to systemic delivery, such as insufficient effects or toxicity if the molecule acts upon an undesired tissue [129]. Several strategies of growth factor immobilization on biomaterials have been pursued, resulting in specific growth factor release profiles [130]. Indeed, non-covalent immobilization generally leads to diffusion- or swelling-controlled release of the molecule, whereas when the growth factor is covalently immobilized on the matrix a chemical/enzymatic reaction is responsible for its release [129]. Furthermore, systems in which growth factors and proteins have been physical encapsulated into the scaffolds have been also developed, making the biomaterial the delivery system [131]. Specifically referring to bone tissue regeneration, the most studied molecules are BMPs (bone morphogenetic proteins), TGFβ (transforming growth factor beta), VEGF (vascular endothelial growth factor) and PDGF (platelet-derived growth factor) (Figure 1). Recently, Caballero Aguilar and co-authors published an elegant review describing the growth factor loading concentration, delivery and kinetics of release in vivo [132].

BMPs belong to the superfamily of TGFβ and have roles in the processes of chemotaxis, mitogenesis and the osteogenic differentiation of mesenchymal stem cells, and promotion of angiogenesis [133]. Specifically, BMP-2 and BMP-7 have already been approved in clinics by the FDA [134]. Recombinant BMP-2 improves the effect on bone formation of MgHA scaffolds, supporting the osteogenic and angiogenic effect of Mg [135]. Even though the addition of BMP-2 to polymers is not sufficient to support bone repair as well as the addition of HA [136], when BMP-2 is combined with HA an intensive mode of mineralized bone formation occurs [137]. Additionally, BMP-9 has been used in order to functionalize a composite scaffold, revealing its ability to promote the differentiation of BM-MSCs into osteoblasts in vitro and to enhance bone formation in vivo [138]. VEGF is produced by many cell types and its activities include angiogenesis and bone formation [139]. PDGF is a potent mitogen able to induce angiogenesis and it also has the ability to improve bone regeneration increasing the surrounding vasculature. Since conflicting results have been reported regarding the effects of PDGF administration for bone healing, a combination of osteogenic factors with angiogenic signals in a spatiotemporal delivery defined system might be the best strategy for bone regeneration [140,141]. The hypothesis involving the co-delivery of growth factors to achieve the best healing stimulation has also been approached [92]. Kirby et al. developed a PLGA based delivery system within a PCL scaffold, including VEGF, PDGF and BMP-2 into PLGA microparticles. Even if PDGF and VEGF did not affect the bone mineralization, they increased the vascularity, an essential event for tissue regeneration [142]. Many other molecules have been studied in order to promote the osteointegration and osteogenesis of scaffolds, such as irisin [143] and platelet gel [144].

## 3. Bone Regeneration: Cellular Component

The most significant tissue engineering strategy for regeneration is the development of a bio-scaffold in which a biomaterial is colonized by cells. Several cell types might be used for the development of a bone construct including osteoblasts, embryonic stem cells (ESC), induced pluripotent stem cells (iPSC) and mesenchymal stem cells. Osteoblasts represent an autologous source of cells that can be obtained from a bone biopsy of the patient, but they are present in limited numbers, with low proliferative potential. ESCs are pluripotent cells derived from the blastocyst inner mass and for that reason they are suitable for regenerative medicine, but their application is still debated due to the risk of teratoma development, immunologic incompatibility and ethical concerns. iPSCs have been generated by engineering manipulation of somatic cells to overcome the abovementioned concerns regarding ESC and have shown interesting features, such as their differentiation potential and autologous source; however, their genetic manipulation has led to doubts for their clinical application [145]. Finally, great regenerative potential for bone tissue undoubtedly lies in mesenchymal stromal cells. Indeed, during normal bone healing, following an inflammatory response, there is a mesenchymal and angiogenic activation phase [146,147]. MSCs play an essential role by becoming bone-forming osteoblasts and chondrocytes that then undergo endochondral ossification [146] and, thus, seem to be more suitable than ESC, iPSC, and osteoblasts for bone tissue engineering [145]. Even though MSCs were initially engaged in regenerative medicine because of their multipotency, other concepts have emerged regarding their therapeutic potential. Indeed, MSCs show several attractive features beyond their plasticity, such as their tropism that makes them able to migrate and home into injured sites in response to specific signals [148]. Moreover, MSCs are recognized as the most promising cells for allogenic cell therapy due to both their immunomodulation ability and immunological escape; also making them interesting for treatment of diseases such as graft-versus-host and autoimmune diseases [127]. In the specific context of bone tissue regeneration, this aspect is very important due to the tight relationship between bone and immune cells, as mentioned above. Indeed, on the cell surface, MSC express low levels of class II major histocompatibility complex (MHCII) and CD40, CD40L, CD80, and CD86 molecules that have a costimulatory effect [149]. Their immunomodulation is mediated by both direct cell-to-cell interactions and paracrine signals, such as cytokines, chemokines and extracellular vesicles [127,150]. They can participate in both innate and adaptive immunity. The ability to modulate B and T cell activities has been an object of intensive investigation in recent years. They are able to suppress the B and T cells response, for example, inducing apoptosis through IDO (indoleamine 2,3-dioxygenase) [151,152]. MSC can also modulate the innate immune system through crosstalk with NK (natural killer) cells, monocytes, macrophages, dendritic cells and neutrophils [127]. However, as viable cells, their immunomodulatory activity is in turn modulated by the milieu. Indeed, it has been demonstrated that pro-inflammatory and anti-inflammatory microenvironments affect the MSC response and secretome release [153]. Therefore, in order to enhance the therapeutic efficacy of MSC, some conditions have been explored to manipulate their secretory profiles [127]. Among the preconditioning methods, hypoxia is used to modulate their immune phenotype, and priming with immunomodulatory factors [127]. MSC were isolated for the first time from the bone marrow, but the harvesting from this source is already invasive enough. For experimental approaches, the most accessible source of MSC is adipose tissue, from which it is possible to isolate about 500 times more cells than bone marrow tissue [154]. However, both sources have a great stemness ability. The isolation of MSC from fetal (including placenta), amnion, umbilical cord, and cord blood tissues allows one to obtain cells with a higher proliferative rate, life span and differentiation potential compared to MSCs derived from adult sources [155,156,157,158,159]. Moreover, the immunomodulatory potential is also influenced by the source of MSC. It has been demonstrated that adipose-derived MSCs can exert more immunomodulatory effects than bone marrow-derived MSCs; on the other hand, umbilical cord-derived MSCs show better immunological escape and, thus, there is minimal risk of an allogenic immune response [127]. Therefore, the choice of MSC source manipulation will depend on the individual’s specific research and/or clinical application [160].

Several studies have focused on enhancing the regeneration of bone by applying MSCs for both cell therapy and tissue engineering strategies [160,161]. MSC-based therapy includes bone marrow transplantation and the administration of MSC expanded by in vitro culture [162]. MSC therapy has been evaluated in several clinical applications, including the healing of bone fractures, non-unions, various jaw bone defects and the prevention of osteonecrosis, revealing its safety and potential efficacy [160,162]. An up to date search revealed 235 ongoing clinical studies, in recruiting and active status, that involve MSCs for several conditions (https://www.clinicaltrials.gov/). Fifteen studies are now using MSCs to treat bone diseases (https://www.clinicaltrials.gov/). However, some limitations have been revealed in MSC therapy, particularly the short survival time after transplantation and the unclear optimal doses and route of administration [162,163]. In MSC-based tissue engineering approaches, MSCs are implanted with a synthetic bone graft to regenerate in situ. The use of scaffolds that facilitate the local delivery of MSC into the bone defects, reduces the risk of ectopic bone formation. A pilot clinical trial demonstrated the efficacy of MSC seeded on a cross-linked serum scaffold for the repair of a maxillary bone defect [164]. Moreover, a multicentric non-comparative trial demonstrated the feasibility and safety of treating non-unions in the tibia, femur and humerus with autologous, expanded MSCs associated with a bioceramic [165]. However, to date, most of the research is predominantly in the preclinical phase. Technically, several methods of scaffold colonization can be pursued. For example, cells can be cultured on the previously created scaffold [166]; another method is represented by the simultaneous deposition of cells and biomaterial, as demonstrated by Cidonio et al. [167]. Nanocomposite bioinks provide an attractive platform to deliver encapsulated stromal cells producing three-dimensional constructs, that aim to facilitate bone repair and functionality [167]. Sophisticated systems have been developed to reach the biological complexity of native bone tissue structure [168] and to support perfusion and interstitial flow [169]. Qiao et al. reconstructed the native-like structure of osteochondral tissue, designing a three-layered stratified copolymeric scaffold colonized by MSCs with a zone-specifc growth factor delivery. Indeed, this scaffold was made of various elements including: a first layer of superficial cartilage in which MSCs were combined with BMP7 and TGFβ; a second layer of deep cartilage with a scaffold, MSC and TGFβ; a third layer of subchondral bone, in which MSCs were combined with BMP2 [168].

The improvement of bone healing in the presence of MSCs has been demonstrated with metals, ceramics, polymeric and composite materials [167,170,171]. Marcacci and colleagues were able to treat large bone diaphysis defects of four patients by seeding cells isolated from the patients’ bone marrow stroma onto porous HA scaffolds, modeled to reproduce the size and shape of the bone defect. The complete fusion of the implant and bone was observed 5 to 7 months later and the long-term durability (last follow-up after 7 years of surgery) of bone regeneration was observed [172]. For the treatment of a large critical-size bone defect in rabbits, the addition of MSC on silica-coated calcium hydroxyapatite scaffolds allowed for a better degree of osteogenesis than that observed with the scaffold alone. Interestingly, when growth factors were added to the scaffold–MSC construct, the bone healing process was accelerated [173]. Peng et al. demonstrated the efficacy of an MSC-colonized BCP scaffold for the repair of a load-bearing bone defect in a canine femoral head. Indeed, compared to the BCP scaffold alone, BCP with MSC induced greater bone formation, in addition to increasing the strength and compressive modulus in the repair site [174]. Gamblin et al. [175], Humbert et al. [176] and Mebarki et al. [177] described in more detail the use of MSC in combination with ceramics. Alternatively, Desai and co-author reported the efficacy of bone marrow aspirate concentrate (BMAC) injections combined with a demineralized bone matrix in treating tibial non-unions with fracture gaps of less than 5 mm [178]. Regarding the use of MSCs associated with polymers, even though the regenerative potential of PGA and stem cells has been demonstrated in vivo [179], the use of more rapidly degrading materials, such as PLGA, is preferred because they soften with time without interfering with the bone’s regrowth [180]. Harada et al. demonstrated the healing of both critical-size and full thickness femur defects in rats by implanting a PLGA scaffold seeded with MSCs previously differentiated in vitro into cartilage-forming chondrocytes [181]. The chitosan/poly (butylene succinate) scaffolds seeded with human MSC resulted in enhanced integration and significant bone formation in vivo, also validating the osteogenic potential in orthotopic locations in immunodeficient mice [182]. Interestingly, Park et al. demonstrated that a hyaluronate hydrogel colonized with umbilical cord blood-derived mesenchymal stem cells is safe and effective for the regeneration of durable articular cartilage in osteoarthritic knees and also in allogeneic conditions [183]. The fabrication of metal scaffolds with micro- and macro-pores allows for a highly controllable pore size and excellent biocompatibility [184]. These structures are more favorable for the adsorption of serum proteins, promoting the growth of mesenchymal stem cells [184]. However, the corrosion of metals is an intrinsic feature of the materials that still remains [185]. In this context, the composite material of matrigel infiltrated Ti6Al4V scaffolds containing encapsulated MSC represents a valid option to counteract issues linked to metals, albeit with maintenance of the load-bearing properties of Ti [186]. The addition of MSCs to the composite biomaterial poly(3-hydroxybutyrate)/HA/alginate improves the regenerative potential, increasing bone formation in a critical-size defect by approximately four-fold [171]. Kosinski et al. demonstrated the regeneration that occurred after 21 days of transplantation of umbilical cord derived MSCs seeded onto a scaffold of Geistlich Bio-Oss^®^ Collagen in a cranial defect [187]. Although their toxicity to eukaryotic cells is still under investigation [188,189,190], silver nanoparticles could promote the proliferation and osteogenesis of mesenchymal stem cells and improve femoral fracture healing when encapsulated in collagen and used at low concentrations [191]. Indeed, the formation of fracture callus and early closure of the fracture gap may be promoted via multiple routes: (i) chemo-attraction of MSC and fibroblasts to migrate to the fracture site; (ii) induction of the proliferation of MSC; (iii) induction of osteogenic differentiation of MSC via induction/activation of TGF-β/BMP signaling in MSCs [191]. Furthermore, zinc, copper, and imidazole metal-organic framework nanoparticles coated over poly-l-lactic acid nanofibrous scaffolds enhance the osteogenic potential more than poly-l-lactic acid scaffolds [192]. Even though these results open new perspectives in bone tissue engineering and regeneration, other data support the hypothesis that scaffold colonization with MSC cannot always improve bone healing compared to the effects of the scaffold alone or when combined with growth factors [193,194]. Thus, so far, none of the MSC-based products have become the standard of care for bone regeneration [102]. Several challenges remain in MSC-based application, such as those linked to cell therapy (standardization, quality control, GMP manufacturing, logistics, cost, regulatory approval, tissue sources of cells, etc.) and those related to tissue-engineered scaffolds and the co-application of signals promoting cell phenotype and function in vivo. In order to overcome the limits of viable cell therapy and tissue transplantation, a number of investigations have focused on secretome-based approaches, such as studies using extracellular vesicles and cytokines released by MSCs. However, although promising research data have been reported, the translational application has faced problems related to standardization in terms of the manufacturing and analytic processes [160].

## 4. Conclusions

Searching on pubmed for “bone regeneration and MSC” results in more than 2000 journal articles. Limiting the search to clinical studies and clinical trials results in only 15 items being found (https://pubmed.ncbi.nlm.nih.gov/). We conducted a search on https://www.clinicaltrials.gov/ for “bone implant” and “bone scaffolds” excluding withdraw, suspended and unknown status results. We obtained 321 results for bone implants, of which 215 were active, concluded, or terminated and 61 were aimed at bone disease applications. Of these 61, seven already had the results. If the search is conducted for bone implants and MSCs, 28 items are returned. As regards the search for bone scaffolds, 41 studies are in the recruitment phase, active, terminated, or completed. Of them, five are using MSCs, five are based on the treatment of bone diseases, five are for musculoskeletal diseases, two are for bone cysts, two are for bone resorption, one is for alveolar bone loss and two are for periodontal conditions (https://www.clinicaltrials.gov/). On the one hand, these numbers highlight the emerging interest of scientists within the field of bone regeneration and engineering; on the other hand, these findings disclose the need to translate the knowledge obtained from preclinical studies into clinical practice. Indeed, although many strategies to accelerate bone regeneration have been studied, an appropriate treatment that can exogenously regenerate bone tissue with optimum morphology and mechanical properties has not yet been achieved [144]. Tissue engineering approaches for the repair of bone defects have attempted to mimic the natural process of bone healing by delivering cells that are able to differentiate into osteoblasts, growth and differentiation factors and degradable scaffolds to support cellular attachment, migration, and proliferation [195]. The choice of the appropriate material for the scaffold is driven by several factors such as the clinical application, the resorbability, mechanical properties in the case of load-bearing bone, osteo-induction/conduction and osteointegration. However, to date, no single material has all the necessary properties to efficiently resemble the mechanical competent of bone tissue. Among cells used for the scaffold colonization, in order to increase the regenerative potential of the treatment, MSCs have been considered as representing the best option so far. These cells show several advantages beyond their ability to differentiate into osteoblasts, including their capability to orchestrate the healing response by paracrine signaling, their immunological escape ability and their immunomodulation ability. Even though several approaches have been tested, including the combination of different biomaterials, so far none of them have been firmly associated with the treatment of a specific bone defect. Further research is needed to solve the various questions related to the use of engineered scaffolds—including those associated with the mechanics, vascularization and complete substitution with new bone tissue and cell-based tissues—in order to translate the knowledge into clinical practice.

## Data Availability

Not applicable.
